# Economic and Behavioral Influencers of Vaccination and Antimicrobial Use

**DOI:** 10.3389/fpubh.2020.614113

**Published:** 2020-12-21

**Authors:** Caroline E. Wagner, Joseph A. Prentice, Chadi M. Saad-Roy, Luojun Yang, Bryan T. Grenfell, Simon A. Levin, Ramanan Laxminarayan

**Affiliations:** ^1^Department of Bioengineering, McGill University, Montreal, QC, Canada; ^2^Department of Ecology and Evolutionary Biology, Princeton University, Princeton, NJ, United States; ^3^Lewis-Sigler Institute for Integrative Genomics, Princeton University, Princeton, NJ, United States; ^4^Princeton School of Public and International Affairs, Princeton University, Princeton, NJ, United States; ^5^Fogarty International Center, National Institutes of Health, Bethesda, MD, United States; ^6^Princeton Environmental Institute, Princeton University, Princeton, NJ, United States; ^7^Center for Disease Dynamics, Economics & Policy, Washington, DC, United States

**Keywords:** COVID-19, vaccination, antimicrobial, behavior, hesitancy

## Abstract

Despite vast improvements in global vaccination coverage during the last decade, there is a growing trend in vaccine hesitancy and/or refusal globally. This has implications for the acceptance and coverage of a potential vaccine against COVID-19. In the United States, the number of children exempt from vaccination for “philosophical belief-based” non-medical reasons increased in 12 of the 18 states that allowed this policy from 2009 to 2017 (1). Meanwhile, the overuse and misuse of antibiotics, especially in young children, have led to increasing rates of drug resistance that threaten our ability to treat infectious diseases. Vaccine hesitancy and antibiotic overuse exist side-by-side in the same population of young children, and it is unclear why one modality (antibiotics) is universally seen as safe and effective, while the other (vaccines) is seen as potentially hazardous by some. In this review, we consider the drivers shaping the use of vaccines and antibiotics in the context of three factors: individual incentives, risk perceptions, and social norms and group dynamics. We illustrate how these factors contribute to the societal and individual costs of vaccine underuse and antimicrobial overuse. Ultimately, we seek to understand these factors that are at the nexus of infectious disease epidemiology and social science to inform policy-making.

## Introduction

Vaccines are among the most cost-effective health technologies of all time. They have been responsible for the two instances, smallpox and rinderpest, in which an infectious disease has been eradicated (2). By choosing to be vaccinated, an individual protects themself but also protects their community by preventing disease transmission. Although immunizing enough individuals in a community above a critical proportion can help prevent outbreaks, actual vaccination levels tend to fall short of epidemiological goals due to vaccine hesitancy and refusal. Vaccine hesitancy is as old as vaccines themselves, but has gained momentum in recent years due to a growing distrust in science and institutions. One recent impetus for this was the subsequently retracted and discredited 1998 study by Wakefield and coauthors which falsely claimed a link between the measles-mumps-rubella (MMR) vaccine and autism in children (3). Vaccine hesitancy runs the range from doubts about a specific vaccine to a complete rejection of all forms of immunization. It is relevant not just to childhood immunizations but also to adult vaccines including those being developed against the severe acute respiratory syndrome coronavirus 2 (SARS-CoV-2), the virus that causes coronavirus disease 2019 (COVID-19).

According to the United States Centers for Disease Control and Prevention (CDC), national vaccination coverage has remained constant in recent years: 91.9–91.5% for MMR, 91.2–91.0% for varicella, 94.1–94.0% for diphtheria, tetanus, and pertussis (DTaP; ≥3 doses), and 72.6–73.2% for rotavirus from 2013 to 2017 (4), but vaccine coverage in certain states and communities has declined. For example, among children aged 5 months, up-to-date status for recommended vaccines declined from 67.9% in 2019 to 49.7% in 2020 in Michigan (5).

Another important method of disease control is through antimicrobial treatment. However, the effectiveness of antimicrobials has decreased in recent years due to the emergence of antimicrobial-resistant strains. Indeed, resistance genes, such as Enterobacteriaceae-producing extended-spectrum β-lactamase (ESBL), NDM-1, and *Klebsiella pneumoniae* carbapenemase (KPC), are widespread and represent major burdens to public health (6). The emergence of antimicrobial-resistant strains has been driven largely by overconsumption and misuse of antimicrobials, which are additionally associated with altered microbiome communities (7), obesity, and irritable bowel syndrome (8).

Despite the contextual complexity of health behaviors, a recent meta-analysis (9) based on conditioned risk questions found that people are more likely to accept vaccination when they perceive a high risk of contracting the disease when unvaccinated (12 studies, effect size 0.26), greater personal vulnerability to the disease (five studies, effect size 0.24), and greater severity of the disease (31 studies, effect size 0.16). Vaccine refusal arises from underestimated risk of disease or overestimated risk of vaccine-induced adverse effects. Risk (mis)perceptions also contribute to overuse of antibiotics. While a lack of awareness about antimicrobial resistance is associated with high rates of antibiotic use among self-medicated individuals (10), clinicians' misperceptions of antibiotic harmlessness are also associated with higher antibiotic prescribing rates in Emergency Departments (11). Studies such as these raise important questions that we must understand to better tackle both vaccine underuse and antibiotic overuse.

What makes people comfortable with the idea of using antibiotics, while being concerned about vaccination, even when the target population tends to be small children? How are these decisions influenced by perceptions of the benefits of antibiotic treatment or immunization, and perceptions of side-effects associated with these interventions? Why do some people perceive vaccines to be unsafe but think that antibiotics are safe? Are individuals likely to take into consideration the benefits (of vaccination) or costs (of antibiotic resistance) that they create for others as a consequence of their actions? And how influenced are they by social norms or peer groups in their behavior? These concerns have increased relevance in the context of COVID-19, where a potential vaccine or set of vaccines are likely to form part of the long-term strategy to keep the disease in check (12).

In this review, we examine factors that shape vaccine hesitancy and antimicrobial overconsumption and characterize the risk and cost they exert upon individuals and societies. First, in Section 2: Risk Perception, we examine the role of risk perception. In Section 3: Free-Riding and Individual Incentives, we look at the issue of individual incentives and external consequences. In Section 4: Social Norms and Group Dynamics, we examine the types of norms and community histories that govern vaccine- and antibiotic-related health behaviors. In Section 5: Actual Risks, Costs, and Benefits of Vaccine Underuse and Antibiotic Overuse, we estimate the risks and costs to individuals and societies associated with vaccination, vaccine hesitancy, antimicrobial use and antimicrobial resistance. In Section 6: Policy Interventions, we propose a series of policy interventions in an effort to curb vaccine hesitancy and antimicrobial overuse and conclude with Section 7: Conclusion.

## Section 2: Risk Perception

One reason why people underuse vaccines and overuse antibiotics is that their perception of risk differs from what the evidence may suggest. In the United States, the perceived risk of vaccine use in the general population is several orders of magnitude greater than the actual probability of adverse vaccine-associated events (13). Likewise, across several countries, even when antibiotic use is unlikely to have a significant benefit for infection prognosis, a large proportion of patients have been shown to desire antibiotic prescription (14, 15).

Most health-related decisions are made under uncertainty. Game-theoretic models of vaccination behavior predict that individuals will free-ride on the herd protection afforded by others' vaccination status, particularly when the risk associated with vaccination outweighs the risk of infection (16). In fact, vaccine refusal can emerge even when rational assessment favors vaccination, due to bounded rationality of individual decision-makers (17, 18). With imperfect information and limited processing capacity, individuals' perceived risks may deviate from the actual risks in positive or negative directions. This idea also applies to the overuse of antibiotics for viral infections or other situations in which their prescription is inappropriate due to the general misperception that they are harmless. According to prospect theory (19), such misperceptions could arise from a complex combination of cognitive biases such as framing effects, loss aversion, and diminishing sensitivity. Misperception of risks may also explain why individual decisions often deviate from predictions of expected utility theory, or the classic risk-benefit model of health behavior.

A paradox of vaccination is that while disease transmission is eliminated, so is collective memory of the disease, which results in an underestimation of the harm caused by the disease. On the other hand, the immediate risk of adverse effects such as fever, anaphylaxis, and vaccine-mediated infection may cause concerns among parents, particularly considering the underdeveloped and vulnerable nature of the immune systems of newborns. Further, because of omission bias, individuals tend to be more concerned with consequences arising from their actions rather than inactions, and therefore may overestimate the risk of rare adverse effects of vaccines (20, 21).

With antibiotics, there is a perception that they are completely safe. Even when the risk of emerging antibiotic resistance due to antibiotic overuse is acknowledged, individuals tend to assess their personal immediate risk of resistance-related effects to be low (22). The majority of antibiotics prescribed in primary care are done so unnecessarily for viral conditions such as acute cough and diarrhea (23, 24) but based on the perception that they could prevent secondary bacterial infections. The misuse of broad-spectrum antibiotics can lead to the disruption of patients' gut microbiomes (25, 26), obesity, and irritable bowel syndrome (8), as well as the emergence of antibiotic-resistant bacteria which may cause severe infections and spread within a community (27, 28). However, the consideration of such long-term effects is often superseded by the urgency of relieving symptoms, which is associated with antibiotic treatment (29). The problem of antibiotic overuse and emerging antibiotic resistance is multifaceted (30). Risk perceptions of physicians, patients, pharmacists, and livestock owners all have a propensity to drive antibiotic consumption. Policy interventions to encourage judicious use of antibiotics should also consider the multi-scale information flow and interactions among these players.

### Cognitive Biases in Risk Perception

How do people form risk perceptions? In general, this is done using heuristics rather than reflective thinking. One example is the availability heuristic that most people use to assess the likelihood of a catastrophic event based on how readily examples come to mind (31). As mass vaccination successfully reduces the population-level prevalence of an infectious disease, knowledge of the disease also declines over time, leading to underestimations of its severity. While antibiotic resistance is a growing threat to global public health, it is also a relatively recent, rarely reported one. Consequently, a lack of experience with the threat and the relatively low number of reported cases of antibiotic-resistant infections to date may increase the perceived safety of antibiotic use. Another aspect shaping the perceived risk of antibiotic resistance is that it is a “slowly emerging” problem similar to climate change, and the uncertainty surrounding its ultimate severity results in it being assigned a lower priority status compared to more immediate threats (32). Such present bias or discounting also influence medical decisions through physicians feeling pressured to prescribe antibiotics in order to satisfy patients' expectations (33). Fortunately, such heuristics also suggest the possibility of using nudges to influence decision making to improve societal outcomes, as will be discussed in Section 6: Policy Interventions.

Individual health decisions are made based on perceived individual risk rather than societal risks. Parents may choose not to vaccinate their children, despite statistics favoring vaccination, if they perceive their children to be more vulnerable to side effects of vaccination or more resistant to the disease. While epidemiological statistics based on large datasets offer “one-fit-all” recommendations for health decisions, individuals may think such conclusions do not apply to their personal cases (20). For example, older individuals may perceive themselves to be more vulnerable than average to influenza, and thus may vaccinate more accordingly (34). However, in the case of antibiotic use, people are often overconfident that they personally would not be affected by antibiotic resistance despite their awareness of the threat at the community level (22, 32).

The framing of choices also affects how individuals assess decisions and outcomes. For example, Emergency Department clinicians tend to prescribe more antibiotics when they view their possible outcomes as either improving a patients' health or having them remain ill, compared to those who frame this decision as one of balancing the potential harm of therapy vs. a patient's continued illness (11). One potential explanation for the drastically different decision patterns in vaccine underuse and antibiotic overuse is that when deciding whether to vaccinate, people are balancing the risks of vaccine adverse effects vs. disease contraction, similar to the second type of clinicians; while when deciding whether to take antibiotics, patients are more likely to frame the decision as the first type of clinicians do, focusing on the potential positive consequences of antibiotic use while ignore the possible negative outcomes. Combined with the cognitive bias of loss aversion, individuals will refrain from accepting the risk of contracting vaccine-related side effects if the benefit of vaccination is only to keep them healthy with no additional gains to their status quo. In keeping with this notion, omission bias, or the tendency for individuals to feel more responsible for a negative outcome when it is due to their action rather than inaction, further increases the general tendency to avoid risks associated with even very rare events (21). Therefore, when the possible adverse side effects of a vaccine are known, even if the chances of them occurring are very low, individuals tend to be more cautious about actively getting vaccinated compared to the potentially riskier inaction of doing nothing. One reason that omission bias does not seem to affect antibiotic use may once again be due to the misperception that antibiotics are completely safe.

The association between perceived risk and health behavior is not definitive. Other individual or social factors such as emotion and trust could also influence individual decisions (35). Anticipated regret plays an important role in health behavior as people try to minimize regret they expect to experience (36). The stronger anticipation of regret for taking the action of getting vaccinated as well as the weaker anticipation of regret associated with inaction will both encourage vaccine refusal (36). Implementation of certain policies to promote responsible health behavior may actually be destructive for arousing negative emotions about the enforced action, or may reduce individual trust in governments. In an experimental vaccination game, Betsch and Bohm (37) showed that compulsory vaccination increased anger and negative attitudes about vaccination among participants, and decreased vaccination uptake in later voluntary vaccination among the vaccine hesitant individuals.

The social context is crucial in shaping individual trust in authoritative recommendations on vaccine and antibiotic use. Organized resistance to vaccination has a long history dating back to the nineteenth century following the enforcement of the smallpox vaccine in England (38). One interesting characteristic of vaccine critical groups is that they consider trust in others (especially the government) to be passive, and instead associate responsibility and empowerment with the act of individually assessing parental decisions, including those related to vaccination (38). In the case of antibiotic use, the consumer-provider relationship between patients and physicians plays an important role in antibiotic prescription. Physicians often feel pressured to prescribe antibiotics to satisfy perceived patient expectations, which are often overestimated (39). This overestimation can lead to a vicious cycle of escalated antibiotic prescribing practices by leading patients to believe that antibiotics are actually necessary for some self-limiting illnesses (39).

## Section 3: Free-Riding and Individual Incentives

The effects of an individual's use of vaccines and antibiotics extend beyond the first order prophylactic and/or treatment benefits they incur. In the case of vaccination, when uptake is sufficiently high, susceptible portions of a population are protected from infection by the presence of immune individuals, a concept known as herd immunity (40). Antibiotic use results in both positive and negative societal effects, or externalities, that are generally not accounted for by the individual when choosing whether or not to take a drug. On the one hand, when an antibiotic succeeds in curing an individual, society benefits from the positive externality of a reduced chance of transmission of that pathogen (41). On the other hand, treatment with a specific antibiotic results in the selection of pathogen strains that are resistant to that treatment, thereby reducing the expected future usefulness of the drug (41).

In economic terms, the non-excludable and non-rivalrous nature of herd immunity makes it a public good, and as a consequence it is vulnerable to free-riding, or use by individuals who do not contribute to maintaining it (42). More concretely, individuals will choose to vaccinate at a rate that is lower than optimal for society since herd immunity protects them from disease even in the absence of vaccination. Similarly, economic theory predicts that the negative externality of resistance associated with antibiotic use results in individual levels of consumption exceeding those that are societally optimal, since the additional cost of decreased drug effectiveness is not borne by the individual user. These concepts are further complicated by the global nature of pathogen spread and antibiotic resistance, which may alter the incentives for policy implementation in a single geographical region.

### Considerations by Individuals

When the perceived risk of a disease is higher, or in other words the perceived benefits of undergoing a prophylactic intervention against it increase, individual decision makers are more likely to take preventative action (9, 43), a phenomenon known as prevalence-dependent behavior. In some cases, this pattern of behavior may result in counterintuitive health outcomes such as observed increases in cases of HIV and other sexually transmitted infections (STIs) following the widespread use of antiretroviral therapies (ARTs) due to increased risky behavior when the perceived risk of disease is reduced (43). In this sense then, the incredible success of vaccines as a public health initiative may also be related to the decline in their voluntary uptake due to the near elimination of previously common diseases from recent memory.

In the simplest terms, balancing the perceived risk of acquiring a disease is the risk of receiving its medical intervention. In the case of vaccination, although there are undoubtedly real associated risks such as the possibility of the oral polio vaccine (OPV) reverting to a pathogenic form of the virus *in vivo* (44), the incidence of such events is extremely low. On the other hand, despite evidence of lasting effects of antibiotics on human health, elevated levels of consumption reflect a general societal sentiment of safety toward antibiotics. The low perceived risks and high negative externalities associated with antibiotic use are evidenced in a number of theoretical economic studies on individual antibiotic uptake in the presence and absence of a social planner that conclude that antibiotic allocation in an uncontrolled market economy will differ from that of the social optimum (45, 46) and even potentially the Nash equilibrium (47). As an example, despite the absence of rigorous testing regarding the efficacy of using growth promoting antibiotics (GPAs) in broiler chicken production, and even evidence that removal of GPAs may *increase* the net value of the flocks (48), the practice was not banned in the USA until 2017.

The costs of these medical interventions, including not only direct medical costs but also indirect costs such as lost time, also influence individual decision making. In one study, 26.1% of respondents in a state with personal-belief exemptions for vaccination stated that they submitted such an exemption for convenience purposes to enroll their children in school, as doing so was less costly than fulfilling the vaccination requirements (49). Therefore, the cost of vaccination must be maintained at low levels, at least relative to the cost of opting out, to encourage uptake in order to compensate for the associated positive externality of herd immunity. In the case of antibiotics, the choice of drug treatment is often made on the basis of cost-effectiveness (41). This, along with institutional control of drug procurement and the fact that clinical treatment guidelines are typically issued by national public health bodies, results in frequently uniform antibiotic choices for given conditions (41). However, the use of a single drug increases the likelihood that a resistant strain will evolve (41), which consequently decreases its future effectiveness. As a result, in order to preserve cost effectiveness while also minimizing the emergence of resistance, economic models for the extraction of non-renewable resources have been used to study the timing of antibiotic use (50, 51). Another solution may be to simultaneously prescribe a variety of drugs randomized over patients in order to mitigate excessive selection pressure toward a single drug or drug class (41). Overall, since individual users do not bear the cost of the negative externality of resistance associated with antibiotic use, there is a need for policy interventions to adjust the price of antibiotics accordingly.

In addition to the perceived cost and benefits of intervention, there is evidence that individual values and sentiments of social responsibility may shape medical decisions. Previous studies found altruism to be a strong motivator in the decision to obtain a vaccination (52), yet in one study where 69% of participants qualified as pro-social as opposed to pro-self as quantified by a social value orientation score, 89% of participants switched behaviors at least once from vaccination to non-vaccination depending on the conditions of the game and their perceived individual infection risks and vaccination costs (53). Importantly, individual values generally cannot be considered in isolation, and must be evaluated within the context of the social norms relevant to the groups they belong to. For instance, one study found that a stronger motivator than either altruism or free-riding for getting vaccinated was the behavior of bandwagoning, or making a decision in line with that of others (52). This type of behavior is consistent with the notion of an availability cascade, or the self-reinforcing process of collective belief formation within groups to avoid individual reputational harm (49). In this way, it has been suggested that one method of increasing vaccination rates may be to preferentially target individuals who form “hubs” of social networks as a result of the social influence they exert over others (54).

### Considerations Across Hospital and National Boundaries

The transboundary nature of disease spread and emergence of antibiotic resistance have prompted a large number of studies into the conditions for cooperation between decision making bodies and how this affects their individual behavior. At the level of hospitals, the control of hospital-acquired infections (HAIs) would likely result in real economic benefits since hospital stays are typically longer for patients infected with resistant bacterial strains (47). In urban settings where patients are exchanged between numerous facilities, game theoretical studies have concluded that the amount a single hospital will invest in hospital infection control (HIC) is dependent on the proportion of patients potentially carrying resistant bacterial strains as well as the strain transmissibility (55). The same study also found that in the absence of coordination, the number of hospitals who will act selfishly and free-ride on HIC investments of other facilities is expected to grow as the number of hospitals in the network increases (55). Therefore, regional coordination and planning between hospitals is likely essential for controlling HAIs (55). Indeed, targeted HIC interventions such as government subsidies and universal decolonization have shown promise in both theoretical (56) and empirical studies (57), respectively.

This same logic also applies at the global scale, and suggests that a global coordinated response may be necessary for the control of antibiotic resistance (58). In the absence of coordination, countries have the incentive to free-ride off of the vaccination efforts of their neighbors without ensuring that their own coverage levels are at the social optimum (59). This notion has received a lot of attention particularly in the context of disease eradication due to the massive potential gains arising from the eliminated need to maintain vaccination. For instance, it is estimated that the annual global benefit of smallpox eradication is about $1.35 billion (using 1967 as a base year), while the total cost of its elimination from endemic countries between 1967 and 1979 was about $300 million (60). While there are a number of pathogen-specific biological, sociology, and epidemiological reasons that complicate the eradication of a particular disease, global cooperation and the incentives of individual nations are also very important considerations (44, 60–64).

## Section 4: Social Norms and Group Dynamics

Vaccine hesitancy and refusal are prominent in geographical and socioeconomic or religious clusters (65). This suggests that an important feature of vaccine-related behaviors is their propagation at the community level. Indeed, a number of studies have supported the idea that vaccine hesitancy and refusal are social norms. Social norms can be broken down into two categories: descriptive and injunctive. Descriptive norms include behaviors that are performed by community members (i.e., what *is* done), while injunctive norms describe behaviors that receive approval or disapproval from the community (i.e., what *ought* to be done) (66). Social norms principally spread via contagion, and this effect is amplified by homophily (67, 68). Studies from numerous sociocultural contexts have illustrated the influence of vaccine-related norms on individual behavior. These outcomes are explained using the Theory of Planned Behavior, which posits that the performance of a behavior is principally the result of its antecedent intention (69). The intention, in turn, is informed by a social norm.

In a number of cases, the norm of vaccine acceptance has been shown to predict individual behaviors. Agarwal et al. (70) investigated a combination of descriptive and injunctive pro-vaccination norms in the context of college students' vaccination behaviors. They found that four out of the six norms tested showed statistically significant correlation with actual vaccination behaviors (70). A study on Nigerian mothers' acceptance of the Bacille Calmette-Guérin (BCG) vaccine showed that living in a community with pro-immunization activism predicted a more than twofold increase in the odds of BCG coverage (71). A similar study on Tongolese mothers found that the communication of a pro-immunization message by the chief, as well as vaccination by the chief himself (a descriptive norm), led to increases in vaccination by mothers for themselves and their children (72).

However, social norms can have negative effects on vaccine uptake if they are anti-vaccination or more generally anti-medicine and anti-establishment in nature. For instance, Brunson found that within an individual's social network, increases in the number of people recommending non-vaccination were associated with an increased likelihood of that behavior (73). Similarly, a study on homeschooling parents' vaccination behaviors found that more parents disagreed with the statement “friends think I should vaccinate my children” than agreed with it. Further, more parents agreed with the statement “friends think the risks of vaccination outweigh the benefits” than disagreed with it. These statements refer primarily to injunctive norms and their effects were reflected in vaccine coverage: only 38% of parents stated that their children had received all the recommended vaccines (74). In the case of the HPV vaccine, gender norms have led to substandard coverage for men. Clinical trial data for the HPV vaccine had originally come from female subjects, so FDA approval was female-specific. Over time, the HPV vaccination recommendations have maintained a gender discrepancy, creating the sense that HPV disproportionately affects women, when in reality, it affects all genders. The feminization of the HPV vaccine has had undesirable effects on the distribution of uptake between the genders: in 2014, 40% of women and 22% of men completed the vaccine series (75). In addition, studies have documented the effects of paternal beliefs surrounding vaccines on the likelihood that mothers will vaccinate their children (74, 76).

Given the weight of evidence suggesting a link between vaccination norms and behaviors, it is important to understand the motivations underlying norm conformity. These motivations can be different for individuals entering a community (social newcomers) and long-standing community members. It is therefore necessary to consider them separately.

### Anti-vaccination as Capital

#### Case Study: Waldorf (Steiner) School Parents

The vaccination tendencies of newcomers to communities with pre-existing anti-vaccination norms can be understood from studies on Waldorf school parents. Waldorf schools offer alternative forms of education and are thought to account for a significant number of children whose parents file personal belief exemption forms. These forms would allow their children to remain un- or under-vaccinated on the basis of non-religious and non-medical explanations and still attend school (77). Sobo found that Waldorf school parents appealed to the traditions of the school culture, which emphasize “[looking] away from biomedicine.” One of the study's participants was quoted as saying, “the school philosophy actually embraces illness because they believe that when your body has a strong illness, particularly a fever, it precedes a developmental leap in the child.” Parents received significant pressure to follow these traditions (77). Based on the analysis of Waldorf school parents, it appears that social newcomers are motivated by a desire to belong, and they adopt community traditions to do so.

Attwell et al. (78) provide a theoretical framework for understanding these types of motivations. They use Bourdieu's theories of “capital” and “habitus” to analyze vaccination decisions. For instance, they suggest that the induction of parents into non-vaccinating communities, can be seen as a drive to acquire cultural capital in a new social context (78). Reich (79) uses a similar line of reasoning for communities of mothers who see themselves as autonomous actors in relation to their children's' health. They mobilize this social capital to gain validation for their rejection of vaccines (79). Attwell et al. also read the tendency to reject vaccines as the acquisition of symbolic capital, which encompasses behaviors that are seen as a “positive sign” and distinguish the group from another, similar to injunctive norms (78). This adds to previous research on “cultural cognition” —the tendency for individuals to match their ideas to those of the broader community as a way of avoiding cognitive dissonance and building solidarity (80–82). This tendency is accentuated when the idea in question is a distinguishing feature of the group (83). A significant finding from Atwell et al. was that individuals moving from one community to another felt a sense of instability, which was then resolved by conformity to the community's accepted “habitus.” “Habitus” refers to the largely unconscious dispositions that a community shares—in this case, the tendency to reject or accept vaccines (78). Therefore, the vaccination decision can be seen as a source of cognitive resolution among social newcomers when the new community holds acceptance or rejection of vaccination as a social norm.

Two cognitive mechanisms have been implicated in norm-related vaccine hesitancy: omission bias (as previously described) and the credibility heuristic (84). The credibility heuristic refers to the tendency of individuals to evaluate the merit of an argument based on the perceived credibility of the source. Importantly, individuals tend to confer credibility to sources with whom they share an in-group connection (84, 85). These mechanisms are consistent with the framework of social identity theory, which posits that the social context for vaccination decisions puts them outside the realm of individual rationality (86, 87). One particularly pertinent example of a long-standing anti-vaccination group is those believing in complementary and alternative medicine (CAM). This group encompasses an ensemble of communities that believe in an entirely DIY approach to medical care. A study on these communities found that they selectively rely on information about the failures of Western medicine to justify their tendencies, while ignoring information about its utility (88). These biases also highlight the possibility of in-group attachment being channeled into outgroup hate. A study of the ways in which non-vaccinating parents portray the vaccinating mainstream found that they constructed a narrative of lifestyle, health, and decision-making inferiority for vaccinating parents. This narrative helped to further cement their beliefs (88).

### Other Group Dynamics

There are certain groups for which the social norm of non-vaccination has distinct historical origins, and it is worthwhile to consider them separately. Several studies have found that African-Americans refuse vaccines more than other racial groups (89–91). Historical tensions between the medical community and African-Americans have been proposed to account for this tendency to reject vaccines. Mistrust of the medical establishment among African-Americans stems back to the era of slavery, when slaves were used as subjects for medical experimentation without their consent or personal gain. Out of this phase of selective experimentation grew the sense among African-Americans that medical technologies were weapons designed to be used against them. This type of stigma has persisted (92).

Orthodox protestant communities have also been studied for their vaccination decisions. Historically, religious arguments both in favor of and against vaccination have been circulated in orthodox protestant groups. Ruijs et al. (93) distinguish between appeals to tradition and “deliberate” choice in religion-based decision making. In traditions-oriented families, they found little evidence for choice consideration at all. A Bourdieusian analysis of this approach to the decision would theorize that non-vaccination was “habitus” for these families. Among the deliberate choice group, most participants cited personal religious experiences (for instance, praying to God for help in the decision) rather than consultation with religious leaders as the predominant factor in their choice (93). This finding suggests that orthodox protestant communities are structured to prioritize personal experience over the influence of social leaders.

### Norm Effects on Antibiotic Use and Prescription

Within various groups, including the aforementioned CAM community, there exists a belief that Western medical practices are “unnatural” (94). In these groups, the reasoning for rejection of vaccines and antibiotics tends to overlap. Looking at public beliefs about antibiotics, Norris et al. (95) found that for many of their study's participants, aversion to antibiotic use reflected a more general reluctance to take any sort of medications (95). Previous research had elucidated a relevant psychological mechanism: namely, the effect of illness perceptions on help-seeking behaviors (96) (e.g., inquiring about the possibility of taking a course of antibiotics). Beliefs in either ‘holistic’ medical practices or the body's innate power to fight off infections was associated with a decrease in such help-seeking behaviors (97–100), and therefore, likely a decrease in willingness to use antibiotics. However, rejection of antibiotics in general does not appear to be held as an injunctive norm in the same way that vaccine hesitancy can be. More often, norms around antibiotic use lead to overuse, and this trend is pertinent to the discussion of antibiotic resistance. Norms play a role in two ways: by affecting patient expectations and by affecting prescriber approaches.

The tendency for individuals to use antibiotics is related both to their perceptions of antibiotic efficacy and their perceptions of whether antibiotics are needed for their particular ailments. As mentioned in the context of vaccine decisions, the basis on which individuals form these sorts of perceptions is in large part the behaviors of the individuals in their social networks. This is true of other medical interventions as well. For instance, Zikmund-Fisher et al. (101) found that when presented with descriptive norms relating to cancer treatment, study participants took behavioral cues from those norms (101). There is some evidence to suggest that patients take similar social cues in relation to their antibiotic use, but it is limited (102, 103). More research must be done to investigate this connection. However, a significant body of literature has shown a related social connection to antibiotic use: namely, that cultural values affect patient demands for prescription antibiotics. This connection has been studied in Australia (104, 105), England (106), France and Germany (107), Europe in general (99, 100, 108, 109), Egypt (110), and Tanzania (111). In all of these cases, ideas about the physician's role in the physician-patient relationship, the need for antibiotics, the efficacy of antibiotics, and the dangers of antibiotics affected patient demand for prescription antibiotics. Cultural values can also have an impact on the use of non-prescription antibiotics. For example, Widayati et al. (112) found that overuse of non-prescription antibiotics in Yogyakarta City, Indonesia, was attributable in part to the prominent belief that medical consultation is a waste of time (112). Interestingly, notions of self-efficacy were associated with high rates of non-prescription antibiotic use in Lithuania, even when the majority of knowledge about antibiotics was coming from medical professionals (113). In Palestine, similar notions of self-efficacy, combined with a positive attitude toward medications and a lack of public education about antibiotics was associated with high rates of non-prescription antibiotic use (114).

In addition, prescription norms and physicians' own ideas about their roles in relation to their patients can influence trends in antibiotic use. For example, Chan et al. (115), studying a hospital in Singapore, found that junior physicians deferred to the practices of senior physicians, thus setting up a norms-based prescribing pattern in their hospital. This pattern was problematic, because physicians tended to focus on their subjective clinical judgements, as opposed to the hospital's guidelines on antibiotic prescription (115). A similar hierarchical, norms-based prescription structure was found by Papoutsi et al. (116) in their study of doctors-in-training (116). In a comparison of antibiotic use in France and Germany, Harbarth et al. found that one of the primary factors accounting for the greater antibiotic use in France was the difference in prescription norms. In Germany, for suspected cases of respiratory tract infection, diagnostic tests were performed, whereas in France, in the face of diagnostic ambiguity, prescription of antibiotics was the default practice (107). Further, physicians' ideas about the benefits of prescription, which are culturally influenced by the degree of community emphasis on guideline adherence and patient satisfaction have been shown to affect their likelihood of prescribing antibiotics, following the Theory of Planned Behavior (117).

Physicians' ideas about their roles in the physician-patient relationship can also have an impact on rates of antibiotic use. For instance, Butler et al. (118) interviewed physicians on their prescribing behaviors, and one participant who admitted to ignoring the guidelines on antibiotic prescription said, “I'm quite well aware of the lack of firm evidence that antibiotics treat [upper respiratory tract infections] and that in terms of evidence-based medicine we overprescribe antibiotics, but my own view is that I don't really care… your goals at the end of the conversation is for both you and the mother and the baby to be satisfied.” (118) In this case, the physician sees his primary responsibility as his patient's satisfaction. Similarly, Kandeel and colleagues' study on antibiotic use practices in Minya, Egypt, found that antibiotic prescription was significantly associated with the patients' preferences for such treatment (110). Thus, the over-prescription of antibiotics appears to be influenced by a combination of hospital-specific descriptive norms and broader cultural ideas about physician responsibilities.

## Section 5: Actual Risks, Costs, and Benefits of Vaccine Underuse and Antibiotic Overuse

Vaccine hesitancy is a growing issue (119) which poses risks and costs to societies and individuals alike, including increased infection rates, economic costs, and decreased herd immunity. Vaccine hesitancy is facilitated by a number of factors, including the option to obtain non-medical exemptions in several states (120) which increases the likelihood of disease outbreaks. An overwhelming driver of vaccine hesitancy is the belief of adverse reactions that an individual can have to vaccines. To address these concerns, the Vaccine Adverse Event Reporting System (VAERS) (121) was implemented to factually track adverse reactions. Furthermore, the Vaccine Safety Datalink (VSD) actively studies adverse effects post-vaccination, in addition to generally ensuring safety (122).

How does vaccine hesitancy compare to antimicrobial use? Antimicrobial resistance is a worldwide problem that has plagued society since the introduction of antibiotics in the 1940s (123), and has been exacerbated in the present day by antimicrobial overuse. The sustained emergence of resistant pathogen strains results in the need for continued development of more powerful antimicrobials, and current drug discovery efforts are unlikely to be sustainable. Furthermore, it is believed that significant improvements to current antibiotics will remain elusive (124). Motivated by this, in 2015 the WHO enacted the “Global action plan on antimicrobial resistance” (125) in a large-scale effort to curb antimicrobial resistance and develop strategies to address this issue.

In this section, we first examine general societal risks and costs associated with vaccine refusal, along with societal benefits of vaccination, in addition to risks and costs of antimicrobial resistance. Then, we briefly summarize risks, costs, and benefits at the individual-level. Subsequently, to highlight and contextualize these general ideas, we present two contrasting cases studies for vaccination and conclude with a specific example of antimicrobial resistance and its consequences.

### Societal Aspects

#### Vaccination

Perhaps the most obvious societal cost of vaccine underuse is the cost of treating vaccine preventable illnesses (126). This cost takes two forms: actual hospitalization, treatment and mortality, and downstream effects. Oftentimes, the cost of even a single hospitalization far outweighs the cost of immunization. For example, a “successful” infection by Diphteria and Tetanus invariably lead to hospitalization. Whitney et al. (126) estimate that the costs of treating these diseases (i.e. hospitalizations) are about $17,000 and $100,000, respectively, although in 2017 the total costs of treating a single unvaccinated child for Tetanus could exceed $800,000 (127).

It is also important to consider both direct and indirect consequences of vaccine refusal. On a broader scale for the USA, Ozawa et al. (128) estimated that adults lacking immunizations represented an economic cost of about $7 billion annually. This number was reduced to $2 billion for infected vaccinated individuals. Moreover, for vaccination against varicella, Zhou et al. (129) showed that the benefit to cost ratio in the USA is about 4 for a single dose of the vaccine, and about 3 for two doses. Furthermore, Omer et al. (130) found that increased non-medical exemptions in the USA resulted in an increased disease burden of pertussis.

#### Antimicrobial Resistance

In a landmark study, Michaelidis et al. (131) computed the “hidden” societal cost of a single course of antibiotics. These authors focused on costs attributed to antibiotic resistance, and concluded that each course of antibiotics imposes a societal burden equivalent to $13 on average. This cost is non-negligible considering that the actual antibiotic cost to the individual may vary from a few dollars to a few hundred dollars.

It is currently estimated that antibiotic resistance poses a significant societal burden through infection: In the US, infections are on the order of millions (~2 million), leading to several thousand deaths (≥23,000) (132). The societal implications of these infections include not only the costs associated with these high annual infection and death rates, but also the increasing probability of these resistant bacteria infecting susceptible hosts as the number of infected individuals rises. For instance, in a study examining *Salmonella* outbreaks, Varma et al. (133) found that infections with resistant pathogens led to 14% more hospitalizations than infections with non-resistant strains. Despite this, most current societal cost estimates only consider the direct consequences of infection with a resistant bacterial strain, and consequently underestimate their true burden (e.g., surgeries would lead to substantially more infections) (134).

### Individual Aspects

#### Vaccination

Individual risks and costs of vaccination lie almost entirely along two axes. First, as previously discussed, there are quantifiable risks of adverse effects associated with every vaccine, as discussed by Stratton et al. (135). In a study spanning multiple vaccines, Bohlke et al. (136) found a rate of 0.65 cases of anaphylaxis per million doses of vaccines administered. Out of five such cases identified, anaphylaxis did not lead to mortality. The second dominant risk is that of an individual's infection probability (137), although as previously mentioned in the context of herd immunity, this probability, and thus its associated cost are functions of the vaccination decisions of others. If vaccination achieves a high enough threshold such that herd immunity is maintained, then the individual that refuses vaccination will be effectively protected with a very low probability of infection. On the other hand, if herd immunity is not achieved, the probability of infection for an unvaccinated individual depends upon the value of *R*_0_ for the specific disease. For high values of *R*_0_, Susceptible-Infected-Recovered (SIR) disease models (138, 139) predict that nearly all initially susceptible individuals will be infected. Thus, refusing vaccination before such an epidemic will very likely result in infection and any complications that may ensue.

#### Antimicrobial Resistance

Antimicrobial resistance gives rise to many individual risks and costs for the individual. First and foremost, incorrect administration of antimicrobials can enhance selection for resistant pathogens and aggravate infection (140). There are also adverse effects associated with using more potent antibiotics to combat resistant pathogens, such as perturbations to the human gut microbiome (141), which may be correlated with severe downstream consequences ranging from weight-gain to increased susceptibility to other infections and even to cancer (142). Thus, conditional upon successful treatment, infection with pathogens that are resistant to milder antibiotics may have effects that last significantly longer than the actual infection.

### Case Study: Influenza Viruses

Influenza viruses are single-stranded, negative sense, segmented RNA viruses that exert significant yearly seasonal burdens on human populations (143) largely due to rapid evolution of the immunodominant hemagglutinin (HA) surface protein (144). Current vaccination strategies elicit immune responses to exposed HA regions that are known targets of antibodies (145), but only have moderate efficacy (146). To further increase immunity in human populations and reduce the number of necessary vaccines to maintain herd immunity, there are currently significant efforts aimed at developing and understanding the impact of Universal Influenza Vaccines (UIVs) that would provide broad protection across multiple strains for multiple years (147–149).

Influenza vaccine refusal is often shaped by preconceived notions of low effectiveness (150). A meta-analysis of studies pertaining to influenza vaccination and health care workers revealed similar notions, in addition to beliefs of low personal risk associated with actual influenza infection (151). Thus, individuals believe that the risk of adverse reactions due to influenza vaccines far outweighs the protection they provide (152). Yet, recent work indicates that vaccination dramatically decreases actual individual risk, both in children [e.g., (153, 154)] and adults [e.g., (155)].

For individuals that are at low risk of influenza complications, the single most compelling reason for annual vaccination is to increase herd immunity and thus lower the probability of transmission to individuals that are at risk of complications. In general, those at elevated risk include children younger than 1 year of age, adults older than 65, pregnant women, and those with chronic illnesses (156). Despite the current “imperfect” seasonal vaccines that require yearly updating and that have mixed efficacy, their societal impacts have been important. Arinaminpathy et al. (157) estimated both direct protection, i.e., a vaccinated host successfully resisting influenza infection, and indirect protection, i.e., a potential host averting infection due to reduced transmission from vaccinated hosts. These authors concluded that vaccination reduced seasonal influenza burden in the United States by between roughly 10 and 37 million cases. Overall, advantages of UIV would include decreased yearly cases and transmission, as well as a change in the evolutionary dynamics of the influenza virus, which may potentially reduce its future burden (158).

### Case Study: Measles

Measles morbilliviruses are singled-stranded, negative-sense, non-segmented RNA viruses that principally infect school-aged children (159). Measles infections can lead to severe complications or damage to the central nervous system years after infection. Due to long-lasting immunity following immunization, vaccination against measles has been highly successful. For example, following the successful use of the MMR vaccine in the USA, endemic measles has been eliminated (160). Following the publication of a retracted paper on the association of the MMR vaccine with autism, vaccine safety has been the subject of extensive studies that reveal no association with autism (161). Yet, vaccine hesitancy introduces pockets of susceptibility in populations. In conjunction with high transmissibility, this results in a loss of herd immunity and possible measles epidemics. For example, in Washington state, there was recently a measles outbreak (162). These outbreaks impose a significant burden on public health infrastructure, in addition to exerting significant direct and indirect costs on individuals.

The economic consequences of measles vaccine hesitancy can be significant [e.g., see predictions of (163)]. Furthermore, a retrospective modeling analysis following the 2012–2013 outbreak in Merseyside, UK established that this outbreak could have been averted with 11,793 vaccines (182,909 pounds) instead of costing 4.4 million pounds due to infections (164). In addition, there are serious public health risks associated with vaccine hesitancy, including substantially more infections (163) and infection risk for those that refuse vaccines [e.g., for measles risk pertaining to exemptions in children, see (165)]. But the individual risk tied to MMR vaccine refusal extends beyond immediate infection into long-term immunological consequences for children that contract measles. Indeed, immunomodulation by measles infection in children leads to immunosuppression (166). Subsequently, other opportunistic pathogens can infect these immunocompromised children, resulting in increased childhood mortality (166).

### Case Study: Methicillin-Resistant Staphylococcus Aureus (MRSA)

*Staphylococcus aureus* is a bacterial human pathogen that causes skin and blood infections. Perhaps the archetype of antibiotic resistance, *Staphylococcus aureus* is also responsible for significant nosocomial infections. How did methicillin-resistant *Staphylococcus Aureus* (MRSA) emerge? Treatment for this pathogen began through the introduction of penicillin in the 1940s. Yet, the emergence of resistant strains to penicillin led to the use of methicillin to successfully combat this pathogen (167). Eventually, however, resistance to methicillin developed, leading to current MRSA. This “specialization” of resistant bacteria acquiring further resistance illustrates that antimicrobial resistance is not novel. Furthermore, genetic analyses have revealed that these MRSA pathogens are further specializing and becoming vancomycin resistant (167).

At the individual level, the odds of dying following surgery when infection with MRSA is acquired compared to without infection are 11.4:1. In contrast, these same odds compared to infection with a non-resistant *Staphylococcus Aureus* strain are 3.4:1 (168). The individual cost of an MRSA infection extends beyond the direct health implications of such infections. Described as the “twenty-first century lepers” (169), MRSA patients also face tremendous stigma due to fear of contagion. Therefore, the social isolation that results from this stigma may lead to significant psychological harm, substantially increasing the individual burden of a MRSA infection.

What are the societal costs of MRSA outbreaks? Stigmatization can lead to decreased cooperativity and productivity, affecting proper societal functioning. Furthermore, MRSA outbreaks can exert pressure on societal resources. For example, a recent MRSA outbreak in a Finnish hospital facility in 2003–2004 lasted 14 months and had tremendous direct and indirect costs (170).

## Section 6: Policy Interventions

### Direct Manipulation of Cost or Supply of Vaccines and Antibiotics

Several policy proposals to target the overuse of antibiotics and the underuse of vaccines suggest direct manipulation of drug supply or pricing. Among these, extended durations for antibiotic patents have been suggested, which incentivize patent owners to curtail their usage (171). Another proposed solution has been to promote a single buyer for antibiotics, as this actor would have the incentive to take future resistance and drug effectiveness into consideration during their purchasing (171). Pigouvian taxes on antibiotics to absorb the externality of resistance development have also been proposed (47, 58), although a clear disadvantage of such a policy is that it disproportionately affects poor users while doing little to limit the consumption of more affluent ones. In this way then, Hollis and Maybarduk suggest that such a tax may be better applied to industrial and agricultural uses where sensitivity to taxation is more equal (58). Nevertheless, even if reductions in antibiotic use are successful, novel drug classes will need to be developed for sustained treatment options in the future (6). To this end proposals for reimagining the business models for drug development have been proposed (6), as well as incentivizing drug discovery through financial rewards that are delinked from drug prices or volumes (58).

Similarly, it has been suggested that one approach to incentivize vaccination may be through the implementation of fines or rewards (42). However, whether or not such policies would succeed in increasing vaccine uptake are debatable. Indeed, when disease prevalence is high, numerous studies suggest that public uptake of vaccines will also rise, making subsidies irrelevant if they are not properly timed (43). Further, there is evidence of utilization of fines as a means to “buy out” of the action they are designed to incentivize in the first place (42).

### Changes to Medical and Prescriber Practices

Another proposed approach to altering vaccine and antibiotic use is through changes in medical practices and physician prescribing behavior. In terms of antibiotic prescribing, studies have found that interactive, computer-based antibiotic guidelines have been successful at reducing the number of inappropriate drug prescriptions (172). Further, the prescription of an antibiotic has sociological ramifications for both the physician and the patient, such as the conclusion that a diagnosis has been made and that the visit is terminated (172). Therefore, for a patient to feel satisfied without an antibiotic prescription, it is recommended that additional explanations detailing the true medical usefulness of these drugs be provided by the physician (172). Indeed, simple interventions to encourage physician-patient communication about the use and risks of antibiotics were shown to effectively reduce antibiotic prescription by 60% (39). Necessarily, however, such recommendations will need to be adapted to the local country and culture within which they are implemented. For instance, antibiotic use without prescription is common in many low-and-middle-income countries (6), and it will be necessary to ensure that those who need the drugs most are still able to obtain them following any policy changes.

Along a similar vein, the way that medical information is presented can also play an important role in treatment uptake. For example, the erroneous over-identification of human papilloma virus (HPV) as a female-specific disease has disadvantaged males from receiving its vaccine, despite growing evidence of a causal role for this virus in penile cancer, anal cancer, and other conditions men are susceptible to (75). This insufficient vaccine uptake among men is exacerbated by an inadequate uptake of the vaccine among women, and hence the inability to generate herd immunity (75). Furthermore, there is evidence to suggest that the way that medical information is presented plays an important role in the magnitude of the risk perceived by the patient (49). In one of the most basic forms of such an intervention, pneumococcal vaccination rates among high-risk patients were found to increase when simple educational information was provided and patient-physician communication about the vaccine was encouraged (173). Further, several studies have found that individuals perceive a risk as greater when it is presented in terms of a frequency (i.e., a 1 in 10 chance) as opposed to a probability (i.e., a 10% chance) (49). Consequently, one strategy for increased vaccination uptake may be for physicians to also present the risk of non-vaccination in frequency terms (49). Similarly, betrayal aversion, or the emotional reaction associated with an object of trust betraying its implicit promise of protection, has been found to occur less frequently when visual aids are employed to communicate risk, suggesting that this may also be a useful strategy for healthcare workers in discussing vaccination (49).

### Nudging and Influences to Social Behavior

The range of biases shaping the perceived benefits of vaccination have resulted in individuals with a spectrum of opinions, ranging from those entirely opposed to vaccination to those who are vaccine hesitant, i.e., susceptible to the anti-vaccination message but whose preferences are not hard-and-fast (49). Drawing on concepts from behavioral law and economics, a number of recommendations, or nudges, targeted at vaccine hesitant individuals in order to correct predictable errors in risk assessment of vaccination caused by cognitive biases without restricting individual choices have been suggested (49). For instance, similarly to the notion of bandwagoning previously introduced, there is evidence that simply informing individuals that others are choosing to vaccinate can increase their uptake (42). Additionally, receiving concrete information through face-to-face interactions may be more effective at initiating action than abstract information, from say a pamphlet (174). Finally, although the ethics of this form of nudging may be more questionable, one study found that when combined with a specific plan for action regarding how and when to get vaccinated, fear was a successful initiator of increased tetanus vaccine uptake among seniors at Yale University (175).

Modifications to laws and government policies regarding vaccination have also been proposed as nudging tools. Herd immunity could be made an excludable good by restricting the community and social activities unvaccinated children can participate in (42), as was done in Rockland County NY in March 2019 following a large measles outbreak. Alternatively, and perhaps more of a shove than a nudge, it has been suggested that removing all philosophical exemptions from vaccination may be an effective way to reduce free-riding and increase uptake (49). Indeed, Mississippi is the state with the highest vaccination rates in the USA, and is one of two states that do not permit religious or philosophical exemptions (49). However, there are concerns that such a hardline approach may not successfully increase uptake given the likelihood of it facing legal challenges and political pushback, and more subtle options in which vaccination is made the default option have been proposed (49). Further, Chapman et al. (34) found that uptake of flu vaccinations increased when the appointments to receive them were pre-scheduled, and allocation of patients to doctors based on geographical proximity has been proposed as a mechanism to alleviate patient attrition in response to reduced antibiotic prescription (176).

### Educational Campaigns and Media Coverage

Based on the association between risk perception and health behavior, campaigns to increase the perceived risk of non-vaccination or reduce the perceived risk of vaccination may be effective in promoting vaccine uptake. Fear arousal alone through presentation of dramatic narratives or pictures about the danger of vaccine-preventable diseases has been shown to be ineffective at increasing the intention or action to vaccinate (175, 177). However, it has been shown that individuals overestimate the risk or frequency of occurrence of events that are highly publicized (49), and as a result one important way to rectify such a cognitive bias may be through the regulation of media coverage of outlier cases of negative vaccine consequences, perhaps by enforcing that equal coverage of cases of the diseases that they are preventing be broadcast.

In general, social norms-based campaigns have been found to be most effective when they stress a positive injunctive message (178). That is, messages have the greatest positive impact when they express what the individual *should* do: “take your vaccine” instead of “don't refuse your vaccine.” In addition, effective messages convey injunctive rather than descriptive norms: “get your child vaccinated for his or her well-being” instead of “get your child vaccinated because that's what others have done.”

Finally, educational campaigns that emphasize the societal consequences of vaccine refusal and antibiotic overuse and set the social expectations for responsible health behavior could also be effective strategies for encouraging the incorporation of societal risk as part in individual risk calculations. For instance, it is commonly observed that individuals misunderstand their personal role in spreading antimicrobial resistance through their use of antibiotic drugs, and consequently believe that the responsibility for control measures lies uniquely with health organizations (22). In this case, better information regarding the causes of antibiotic resistance and specific instructions on how individuals could contribute to controlling antibiotic resistance would likely prove effective.

## Section 7: Conclusion

In this paper, we reviewed the personal, societal, and economic factors affecting vaccine hesitancy and antimicrobial overuse. These insights are helpful to understand the uptake of a potential vaccine against COVID-19. A variety of misperceptions about risk contributes, in part, to the imbalance of vaccine uptake and antimicrobial use relative to their socially optimal levels of consumption. For instance, individuals may underestimate the risk of a disease because of herd immunity and engage in free riding. They may further overestimate the risk of adverse events from vaccination and underestimate the risk of antimicrobial overuse. This may be particularly relevant in the context of COVID-19, and the importance of rigourous vaccine testing to maintain public trust has been emphasized, despite the simultaneous need for unprecedentedly fast vaccine development (179).

From a policy perspective, complications arise from the multifactorial nature of information flow, involving prescribing physicians, patients, pharmacists, the government, etc…, and the cognitive biases that reinforce misperceptions. Social norms of non-vaccination also appear to push individuals toward vaccine hesitancy and refusal. In communities that hold such norms, social newcomers are highly incentivized to conform as a way of building solidarity and securing their positions. For long-standing community members, on the other hand, non-vaccination may become an unconscious behavior or a deeply-rooted belief. In the latter case, individuals are susceptible to a variety of cognitive biases. Finally, specific racial and ethnic communities may have unique relationships to vaccines for a variety of historical reasons, which will be important for policy makers to consider.

The prevalence of vaccine hesitancy and antimicrobial overuse warrants consideration from policy makers because of the individual, societal, and economic costs that they entail. We investigated several policy interventions aimed at encouraging a shift toward the socially optimal levels of vaccine and antibiotic consumption. These included direct manipulations of the costs of vaccines and antibiotics, changes to prescriber practices, and nudging through modifications of the choice structure or direct regulations on vaccine exemption. NGOs can also engage in nudging through directed campaigns, but the efficacy of these initiatives depends largely on characteristics of the messages that they portray. Based on the successes and failures of previous campaigns, it appears that these messages should be informed by social norms theory. It is critical that any interventions be coordinated between regional actors to match the global nature of pathogenic spread and antimicrobial resistance.

## Author Contributions

The review was drafted by CW, JP, CS-R, and LY with input and supervision from BG, SL, and RL. All authors contributed to the article and approved the submitted version.

## Conflict of Interest

The authors declare that the research was conducted in the absence of any commercial or financial relationships that could be construed as a potential conflict of interest.
